# Deficiency of endothelial FGFR1 signaling via upregulation of ROCK2 activity aggravated ALI/ARDS

**DOI:** 10.3389/fimmu.2023.1041533

**Published:** 2023-03-10

**Authors:** Yue Deng, Xingming Huang, Yan Hu, Weiting Zhong, Hua Zhang, Chunheng Mo, Hongjun Wang, Bi-Sen Ding, Chen Wang

**Affiliations:** ^1^ Peking University China–Japan Friendship School of Clinical Medicine, Beijing, China; ^2^ National Center for Respiratory Medicine, Institute of Respiratory Medicine, Chinese Academy of Medical Sciences, National Clinical Research Center for Respiratory Diseases, China-Japan Friendship Hospital, Beijing, China; ^3^ Key Laboratory of Birth Defects and Related Diseases of Women and Children of MOE, State Key Laboratory of Biotherapy, West China Second University Hospital, Sichuan University, Chengdu, China; ^4^ Beijing Tide Pharmaceutical Co., Ltd., Beijing, China; ^5^ State Key Laboratory of Medical Molecular Biology, Department of Physiology, Institute of Basic Medical Sciences Chinese Academy of Medical Sciences, School of Basic Medicine Peking Union Medical College, Beijing, China

**Keywords:** ALI/ARDS, vascular leakage, inflammation, endothelial cell, FGFR1, ROCK2

## Abstract

Vascular leakage and inflammation are pathological hallmarks of acute lung injury (ALI)/acute respiratory distress syndrome (ARDS). Endothelial cells (ECs) serve as a semipermeable barrier and play a key role in disease progression. It is well known that fibroblast growth factor receptor 1 (FGFR1) is required for maintaining vascular integrity. However, how endothelial FGFR1 functions in ALI/ARDS remains obscure. Here, we revealed that conditional deletion of endothelial FGFR1 aggravated LPS-induced lung injury, including inflammation and vascular leakage. Inhibition of its downstream Rho-associated coiled-coil–forming protein kinase 2 (ROCK2) by AAV Vec-tie-shROCK2 or its selective inhibitor TDI01 effectively attenuated inflammation and vascular leakage in a mouse model. *In vitro*, TNFα-stimulated human umbilical vein endothelial cells (HUVECs) showed decreased FGFR1 expression and increased ROCK2 activity. Furthermore, knockdown of FGFR1 activated ROCK2 and thus promoted higher adhesive properties to inflammatory cells and higher permeability in HUVECs. TDI01 effectively suppressed ROCK2 activity and rescued the endothelial dysfunction. These data demonstrated that the loss of endothelial FGFR1 signaling mediated an increase in ROCK2 activity, which led to an inflammatory response and vascular leakage *in vivo* and *in vitro*. Moreover, inhibition of ROCK2 activity by TDI01 provided great value and shed light on clinical translation.

## Introduction

ALI/ARDS is a severe and dangerous syndrome, clinically characterized by progressive dyspnea and life-threatening arterial hypoxemia, leading to serious mortality (30%-40%) (1). Due to direct (such as pneumonia) or indirect lung injury (such as sepsis) exposure, the morbidity of ALI/ARDS is considerably high, with approximately 10% of all patients in intensive care units across 50 countries suffering from it (2, 3). Currently, the treatment options for this disease are still limited with poor effects.

Disruption of endothelial barrier results in increased inflammation and vascular permeability, which are pathological hallmarks of ALI/ARDS. Pulmonary endothelial cells (ECs) lining the innermost layer of blood vessels act as a semipermeable barrier and perform an important role in maintaining vasculature homeostasis with the assistance of intercellular junctions (tight junctions and adherens junctions) (4). These junctions are facilitated by cytoskeletal microtubules and actin microfilaments under the regulation of multiple signaling pathways (5, 6). Therefore, it is imperative to explore pathways that precisely reconcile endothelial barrier integrity and inflammation, upon which to seek a promising therapeutic target is of great importance.

The FGFR family member FGFR1, a single-pass transmembrane receptor with tyrosine kinase activity, has diverse ligand-receptor specificity and extensive expression in different tissues (7), which leads to the involvement of FGFR1 in a wide range of fields, such as angiogenesis, wound healing and development (8). As the most abundant FGFR of endothelial cells (9, 10), FGFR1 is reported to be responsible for maintaining vascular integrity (11, 12). For instance, FGFR1 activation by recombinant FGF2 treatment decreases permeability in human brain microvascular endothelial cells challenged by oxygen-glucose deprivation/reoxygenation (10) and traumatic brain injury (13). Moreover, another study reveals that FGFR1-dependent recombinant FGF21 protects against blood brain barrier (BBB) leakage (14). On the other hand, FGFR1 has been shown to be actively involved in endothelial repair after vascular injury (15). It serves as a critical mediator in glycocalyx reconstitution of the pulmonary endothelial surface layer during sepsis (10). Indeed, FGFR1 is suppressed in sepsis (10, 16), and an increase in FGFR1 expression attenuates pulmonary inflammation in ventilator induced lung injury (16). However, understanding of the mechanism underlying FGFR1 signaling in the regulation of vascular permeability and inflammation in LPS-induced ALI/ARDS remains incomplete.

Rho-associated coiled-coil–forming protein kinases (ROCKs), downstream effectors of the small GTPase RhoA, play a central role in regulating actin cytoskeleton dynamics by phosphorylating multiple downstream substrates, including myosin light chain (MLC), myosin phosphatase-targeting subunit 1 (MYPT-1) and Ezrin/radixin/moesin (ERM) (17). They function in regulating various cellular functions, such as cellular contraction, proliferation, migration and differentiation (18). ROCKs have two isoforms, ROCK1 and ROCK2, which share 64% overall identity in their primary amino acid sequences (17). Enhanced ROCK activity upon stimulation by inflammatory mediators, such as thrombin, LPS and TNFα, strongly disrupts the vascular barrier (19) and is deeply involved in ALI/ARDS (20). A ROCK2-specific role in monocytic migration, monocyte adhesion toward endothelial cells (21) and vascular permeability (22) has been reported. Considering the potential protective effect of FGFR1 signaling in vascular function, we concluded that deficiency of endothelial FGFR1 by activating ROCK2 aggravated lung injury and that inhibition of ROCK2 activity was considered to be a prospective insight for prevention and treatment of ALI/ARDS.

## Materials and methods

### Experimental animals

Male wild-type (WT) C57/BL6J mice, 8 weeks of age and weighing 22-24 g, were purchased from the Model Animal Research Center of Nanjing University. *Fgfr1* ^loxP/loxP^ mice were crossed with VE-cadherin-Cre^ERT2^ mice to establish VE-cadherin-Cre^ERT2^ *Fgfr1* ^loxP/loxP^ (*Fgfr1* ^iΔEC/iΔEC^) mice, which were identified by genotyping. All mice were housed in a specific pathogen-free (SPF) animal room named the Experimental Animal Center of West China Second Hospital in accordance with the guidelines of the National Institutes of Health. All of the animal experiments were approved by the animal care and were performed in accordance with the guidelines outlined by the committees of West China Second University Hospital, Sichuan University. After the mice were treated intraperitoneally with tamoxifen (100 mg/kg) for 6 days and interrupted for 3 days after the third dose, EC-specific deletion of FGFR1 was induced. WT mice were injected intratracheally with 10 mg/kg LPS or saline, while *Fgfr1* ^iΔEC/iΔEC^ mice and their control littermates *Fgfr1* ^loxP/loxP^ mice were injected intratracheally with 2 mg/kg LPS to maintain their survival. Different doses of LPS were dissolved in 50 μl of sterile saline, and all groups received a volume of 50 μl. The lung tissues were harvested after 24 h. The mice were pretreated orally with the selective ROCK2 inhibitor, TDI01, (Beijing Tide Pharmaceutical Co., Ltd.) for 3 days before LPS injection.

### Adeno-associated virus transduction

The adeno-associated virus Vec (AAV Vec) was manufactured by Hanbio Biotechnology (Shanghai, China). To achieve endothelial-selective gene knockdown, sequence encoding shROCK2 or negative control sequence (NC) was constructed under *Tie1* promoter and expressed in AAV, resulting in AAV Vec-tie-shROCK2 and AAV Vec-tie-shNC. The virus titer was 1.0×10^12^ μg/ml. *Fgfr1* ^iΔEC/iΔEC^ mice were injected intratracheally with AAV Vec expressing ROCK2-specific shRNA and control shRNA (**Table S1**) and then LPS 1 month later.

### Isolation of mouse pulmonary ECs

Fresh mouse lung tissues were washed twice with cold PBS, minced and incubated in a digestive mixture (1 mg/ml of collagenase I and 1 mg/ml of dispase II in DPBS) on an orbital shaker at 37°C for 30 min. The cell suspension was then diluted with the same volume of cold DPBS, filtered through cell strainers and centrifuged. The cells were then washed once with DPBS and treated with red blood cell lysis reagent for 10 min, and washed and then centrifuged. For EC (CD31^+^ CD45^-^) isolation, dynabeads were washed three times with 1 ml of cold MACS wash buffer (2 mM EDTA, 0.1% BSA in DPBS) and incubated respectively with CD45 antibody (BD) and CD31 antibody (BD) at 4°C for 4 h. The dynabeads were then washed three times with MACS wash buffer. The cell deposits were resuspended in 300 μl of MACS wash buffer. Two hundred microliters (200 μl) of Dynabeads-CD45 antibody conjugate (200 μl for 2×10^7^ cells) was added to the cell suspension and then incubated at 4°C for 45 min on a rotator. After incubation, beads bound to CD45^+^ cells were captured by a magnet, and the supernatant was transferred to a tube containing Dynabeads-CD31. The Dynabeads-CD31 antibody conjugate was incubated with the collected supernatant at 4°C for 45 min on a rotator, and beads with CD31^+^ CD45^-^ cells were captured by a magnet. Beads with CD31^+^ CD45^-^ cells were washed three times with cold MACS wash buffer and then prepared for RNA or protein isolation.

### RNA-sequencing

Total endothelial RNA from mouse lungs sorted by dynabeads was isolated with TRIzol reagent according to standard protocols and then sent to Beijing Novo Gene Company for RNA-sequencing analysis. Total amounts and integrity of RNA were assessed using the RNA Nano 6000 Assay Kit of the Bioanalyzer 2100 system (Agilent Technologies, CA, USA). Transcriptome libraries were constructed and quantified by Qubit 2.0 Fluorometer. After the library was qualified, the target amount of data was sequenced by the Illumina NovaSeq 6000. Sequenced reads were aligned to the Mus musculus reference genome (GRCm38/mm10) with HISAT2 (v2.0.5). FeatureCounts (v1.5.0-p3) was used to count the read numbers mapped to each gene. Differential expression analysis was performed using the DESeq2 R package (1.20.0). Gene set enrichment analysis (GSEA) was implemented by the clusterProfiler R package (3.8.1).

### *In vivo* vascular permeability assay

To measure vascular permeability in response to LPS challenge, dextran (70 kDa) and Evans blue dye (EBD) staining were used. In brief, the mice were injected with 50 µl of FITC-dextran or AF555-dextran (50 mg/ml) *via* tail vein 23.5 h after LPS injection. After 30 min, the whole lung tissues were harvested and perfused through the trachea with 200 μl of 50% OCT and immersed in 4% paraformaldehyde at 4°C for 8 h. Each lobe was embedded in OCT at -80°C for frozen sectioning. Then, 7-µm-thick sections were cut and stained with DAPI. The fluorescence of each group was measured by FV3000 (Olympus) and LSM980 microscope (Zeiss). The mice were injected *via* tail vein with 100 µl of 1% EBD 23 h after LPS injection. One hour later, the mice were sacrificed and the lung vasculature was flushed with 10 ml of PBS per mouse through the right ventricle to remove intravascular dye. The whole lung tissues were homogenized in 1.5 ml of formamide and incubated at 60°C for 48 h. The homogenates were centrifuged at 12000×g for 30 min, and the absorbance of the supernatant was measured at 620 nm in a 96-well plate.

### Histology

After collection, the lungs were fixed in 4% paraformaldehyde, maintained for 24 h, embedded in paraffin and cut into 5-μm-thick sections. The sections were stained with hematoxylin and eosin before microscopic histological examination. Pictures were taken by Pannoramic MIDI. The severity of lung injury was scored based on the American Thoracic Society (23).

### Measurement of relative mRNA expression

Total RNA was extracted from lung tissues and cells using TRIzol reagent according to the standard protocol and then reverse-transcribed into cDNA. RT-PCR was performed using the CFX96™ Real-Time system (Bio-Rad). Relative *IL6*, *IL1β*, *IL10*, *TNFα* and *Fgfr1* mRNA expression was assessed. The target gene transcript levels were determined and normalized to the housekeeping gene *Gapdh* using the ΔΔCT method. The primer sequences were shown in **Table S2**.

### Immunofluorescent staining

For immunofluorescent staining, lung tissues were frozen in OCT and cut into 6-μm-thick sections. Cryopreserved sections were incubated with antibodies against mouse VE-cadherin (R&D Systems), FGFR1 (Sigma-Aldrich), ROCK2 (Abcam), ROCK1 (Abcam), MLC2 (CST), ERM (CST), pROCK2 (Abcam), pROCK1 (Abcam), pMLC2 (CST) and pERM (CST) supplemented with 10% normal donkey serum/1% BSA/0.1% Tween 20 at 4°C overnight, followed by incubation with fluorophore-conjugated secondary antibodies at 37°C for 1 h (Jackson ImmunoResearch). Specimens were stained with DAPI and sealed. Images were captured by an LSM980 microscope (Zeiss).

### Cell culture

HUVECs were isolated from the human umbilical cords of healthy volunteers following informed consent and ethics committee approval. They were cultured in endothelial cell basal medium-2 (CC-3156, Lonza) containing 100 U/mL of penicillin and 100 µg/mL of streptomycin (Sigma-Aldrich) at 37°C in a humidified 5% carbon dioxide atmosphere. TNFα (Novoprotein), TDI01 (Beijing Tide Pharmaceutical Co., Ltd.) and azd4547 (MCE) were used to treat HUVECs.

### Monocyte adhesion assay

HUVECs were cultured in 12-well plates. THP-1 cells were suspended (1 × 10^6^ cells/mL) and stained with Hoechst 33342 (Beyotime) at 37°C for 10 min. THP-1 cells were washed three times with PBS to wash off unlabeled THP-1 cells and then plated in HUVECs for a 4-h co-incubation in a 5% CO_2_ atmosphere at 37°C. To remove the unadhered THP-1 cells, HUVECs were washed with PBS three times. After that, adhered THP-1 cells were captured by Carl Zeiss Microscopy GmbH and counted from three randomly selected areas.

### *In vitro* detection of permeability in HUVECs

Permeability of endothelial monolayers *in vitro* was determined by FITC-dextran (3 kDa). HUVECs (1×10^5^ cells/100 μl/well) were plated on the upper chamber of the transwell insert on top of the Matrigel-coated transwell filters (3 μm pore size). After cells adhere (60 min after plating), 200 μl of EBM-2 was added to the upper chamber and 1 ml was added to the lower chamber (in the 24-well plate). After incubating at 37°C for 24 h, the procedure of HUVEC plating was repeated and then incubation was performed for an additional 24 h at 37°C. FITC-dextran was added to the lower chamber to a final concentration of 10 μg/ml. Ten microliters (10 μl) of aliquots of media were removed from the upper chamber and diluted in 90 μl of water/well in a 96-well plate at various time intervals (0, 5, 15, 30, 45, 60 and 120 min after treatment). The fluorescence intensity was measured using a 96-well plate fluorimeter (PE EnVision 2015) with excitation at 485 nm and emission at 535 nm.

### Western blotting

Total protein was extracted from HUVECs and lysed in RIPA buffer containing phosphatase inhibitor cocktail (Bimake) and protease inhibitor cocktail (MCE). Proteins were added to the wells of an SDS-PAGE system, separated by 10% SDS–PAGE and transferred to a PVDF membrane. After blocking in 5% milk for 1 h at room temperature, the membrane was incubated with β-actin (Servicebio), FGFR1 (CST), ROCK2 (Abcam), ROCK1 (Abcam), ERM (CST), MYPT1 (CST), pROCK2 (Abcam), pROCK1 (Abcam), pERM (CST), pMYPT1 (Invitrogen), GAPDH (Servicebio), ICAM1 (Servicebio) and VCAM1 (Abcam) at 4°C overnight and then incubated with horseradish peroxidase (HRP)-conjugated goat anti-rabbit or mouse IgG at room temperature for 1 h. Bands were visualized using the ChemiDoc Imaging System (TOUCH IMAGER™).

### siRNA knockdown

HUVECs in culture were transfected with human FGFR1 siRNA (siFGFR1) and control siRNA (siNC) (GenePharma). The transfection reagent Lipofectamin™ RNAiMAX (Invitrogen) was used. siRNAs were used at a final concentration of 10 nM. The siRNA sequences are shown in **Table S3**.

### Statistical analysis

Data analysis was carried out using the GraphPad Prism 8 software. Statistical analysis of differences between the two groups was performed using the unpaired Student’s t test. p values < 0.05 were considered statistically significant.

## Results

### Pulmonary endothelial FGFR1 expression was decreased in LPS stimulated ALI/ARDS

**Figure 1:**
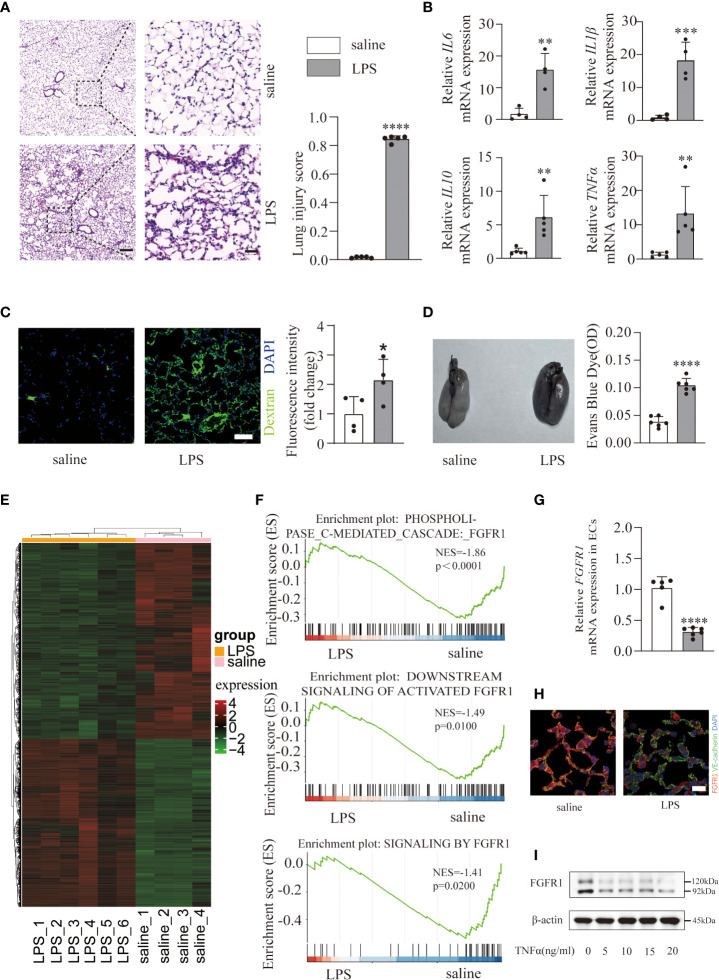
An LPS-induced ALI/ARDS mouse model was constructed and endothelial FGFR1 was decreased in the LPS group. Mice were treated with saline and LPS (10 mg/kg) intratracheally for 24 h. Representative lung sections were stained with hematoxylin and eosin and lung injury scores were quantified **(A)**, n=5 per group. Scale bars: 200 µm (left) and 40 µm (right). Total RNA was isolated from saline- and LPS-treated mouse lung tissues. Relative mRNA levels of inflammatory cytokines (*IL6*, *IL1β*, *IL10*, *TNFα*) were significantly increased in mice treated with LPS compared with saline-treated mice, as determined by RT-PCR analysis **(B)**. n=4 or 5 per group. Images of FITC-dextran were costained with DAPI and quantified by the mean fluorescence intensity of FITC-dextran **(C)**. n=4 per group. Scale bars: 50 µm. Whole lung tissues were stained with EBD-stained lungs (left) and EB content in the lungs (right) was quantified **(D)**. n=6 per group. A gene expression heatmap is shown, and hierarchical clustering was based on 2713 upregulated genes and 3167 downregulated genes between lung ECs intratracheally treated with saline and LPS **(E)**. n=4 or 6 per group. Gene set enrichment analysis of differentially expressed genes in the saline and LPS groups suggested decreased endothelial FGFR1 in the LPS group **(F)**. The relative mRNA expression of pulmonary endothelial *FGFR1* in LPS-treated mice was examined **(G)**. n=5 or 6 per group. FGFR1 was costained with VE-cadherin in the lung sections. Red indicates FGFR1, green indicates VE-cadherin **(H)**. n=4 per group. Scale bars: 20 µm. HUVECs were treated with 0, 5, 10, 15 and 20 ng/ml of TNFα for 12 hours. Cell lysates were analyzed by western blotting with FGFR1 and β-actin antibodies **(I)**. Each bar represents the mean ± SD; *p < 0.05, **p < 0.01, ***p < 0.001 and ****p < 0.0001.

To investigate the underlying mechanism of ALI/ARDS progression, a mouse model was constructed *via* intratracheal instillation of LPS (10 mg/kg) in WT mice. Histological analysis of lung tissues showed marked pulmonary edema and patchy neutrophil infiltration in the LPS group (**Figure 1A**) compared with the saline group. In addition, the lung injury scores (**Figure 1A**) were quantified and showed significant differences, indicating the presence of severe inflammation. Undoubtedly, LPS triggered a severe inflammatory response supported by excessive accumulation of inflammatory cytokines (*IL6*, *IL1β*, *IL10*, *TNFα*) mRNA expression (**Figure 1B**) in lung tissues. Consistent with previous studies (24), we observed a damage to the pulmonary vascular barrier in ALI/ARDS lungs. By measuring and quantifying the interstitial accumulation of the intravenously injected plasma protein tracer FITC-dextran and EBD (**Figures 1C, D**), mice treated with LPS exhibited significantly higher amounts of FITC-dextran and EBD, suggesting vascular barrier disruption. Overall, we successfully constructed an ALI/ARDS mouse model. Since pulmonary endothelial cells function as a semipermeable cellular barrier and play a critical role in the preservation of vascular integrity and inflammation, we performed RNA-seq on pulmonary ECs isolated from the saline and LPS groups to elucidate the transcriptional changes. Hierarchical clustering (**Figure 1E**) of 5880 significantly differentially expressed genes (adjusted p ≤ 0.05; absolute fold change ≥ 1) after LPS treatment revealed a huge transcriptional shift, with 2713 upregulated genes and 3167 downregulated genes. To gain further insight into the key genes that maintain pulmonary vascular integrity, we performed a Gene set enrichment analysis and found a significant decrease in FGFR1-associated signaling pathways (**Figure 1F**). To further verify whether FGFR1 is downregulated in ECs of ALI/ARDS lungs, we confirmed an obvious decrease (**Figures 1G, H**). Next, we investigated FGFR1 expression in HUVECs challenged with different doses of TNFα. The results showed that TNFα induced a significant reduction in FGFR1 in HUVECs (**Figures 1I**; **S1**). Taken together, pulmonary endothelial FGFR1 expression was decreased in LPS-stimulated ALI/ARDS.

### Deficiency of endothelial FGFR1 aggravated inflammation and pulmonary vascular leakage in ALI/ARDS

**Figure 2:**
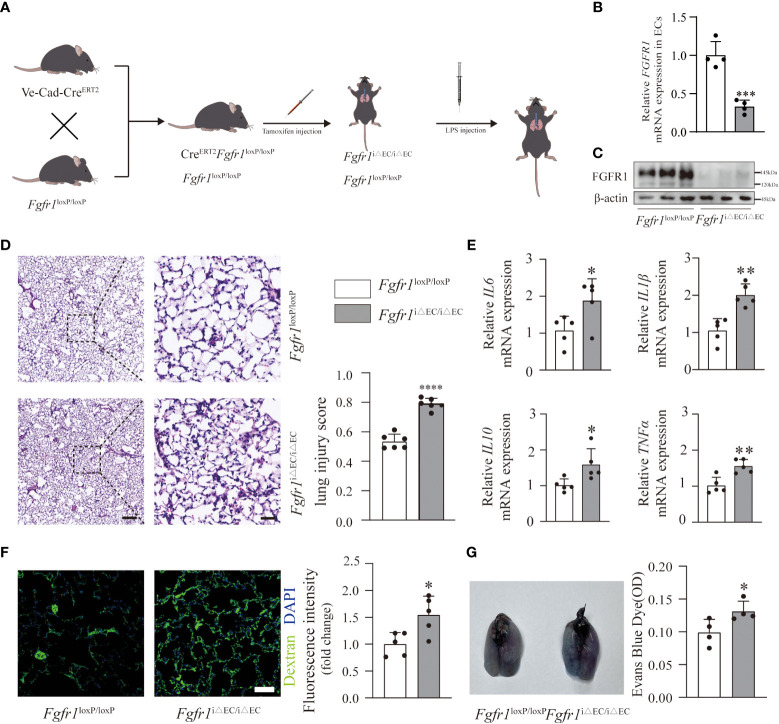
Deletion of FGFR1 in ECs aggravated the severity of inflammation and vascular leakage. *Fgfr1* ^iΔEC/iΔEC^ mice and *Fgfr1* ^loxP/loxP^ mice were intraperitoneally injected with tamoxifen. After LPS injection, ALI/ARDS was established **(A)**. Knockout efficiency of endothelial cell-specific deletion of FGFR1 is shown. RT-PCR analysis of endothelial *FGFR1* mRNA in *Fgfr1* ^loxP/loxP^ mice and *Fgfr1* ^iΔEC/iΔEC^ mice **(B)**. n=4 per group. Western blotting analysis of endothelial FGFR1 in *Fgfr1* ^loxP/loxP^ mice and *Fgfr1* ^iΔEC/iΔEC^ mice **(C)**. n=3 per group. Lung sections were examined for signs of inflammation after hematoxylin and eosin staining and were scored for lung injury **(D)**. n=6 per group. Scale bars: 200 µm (left) and 40 µm (right). Significantly higher lung injury scores **(D)** and higher relative mRNA levels of inflammatory cytokines (*IL6*, *IL1β*, *IL10*, *TNFα*) **(E)** suggested aggravated lung inflammation in *Fgfr1* ^iΔEC/iΔEC^ mice. n=5 per group. Vascular permeability was assessed by extravasation of FITC-dextran **(F)** and EBD **(G)**. Representative images of immunofluorescent staining and quantification of the mean fluorescence intensity of FITC-dextran are shown **(F)**. n=5 per group. Scale bars: 50 µm. Representative images of the gross appearance of EBD-stained lungs and quantification of EBD content in the lungs are shown **(G)**. n=4 per group. Each bar represents the mean ± SD; *p < 0.05, **p < 0.01, ***p<0.001 and ****p < 0.0001.

To address the role of endothelial FGFR1 in inflammation and pulmonary vascular permeability, we conditionally knocked out FGFR1 in the ECs of adult mice (*Fgfr1* ^iΔEC/iΔEC^ mice) (**Figure 2A**) and confirmed the knockdown efficiency (**Figures 2B, C**; **S2**). We hypothesized that the deletion of FGFR1 in ECs would aggravate the progression of ALI/ARDS. To reduce the mortality rate in *Fgfr1* ^iΔEC/iΔEC^ mice, we adjusted the dose of LPS to 2 mg/kg for further experiments (**Figure 2A**). As expected, significantly more infiltration of neutrophils and red blood cells was observed in *Fgfr1* ^iΔEC/iΔEC^ mice compared to *Fgfr1* ^loxP/loxP^ mice, not only in the alveolar space but also in the interstitial space. Moreover, higher lung injury scores reflected aggravation in *Fgfr1* ^iΔEC/iΔEC^ mice (**Figure 2D**). We also examined the inflammatory response as assessed by the relative mRNA expression levels of inflammatory cytokines. There was markedly increased *IL6*, *IL1β*, *IL10* and *TNFα* mRNA expression in *Fgfr1* ^iΔEC/iΔEC^ mouse lung tissues, suggesting an escalation of inflammation (**Figure 2E**). In parallel, *Fgfr1* ^iΔEC/iΔEC^ mice showed more severe FITC-dextran and EBD extravasation (**Figures 2F, G**). Consistent with previous studies (11, 12), inhibition of FGFR1 actually resulted in the loss of vascular integrity, leading to inflammation and vascular leakage.

### Endothelial ROCK2 was activated in ALI/ARDS model and functioned as a downstream of FGFR1

**Figure 3:**
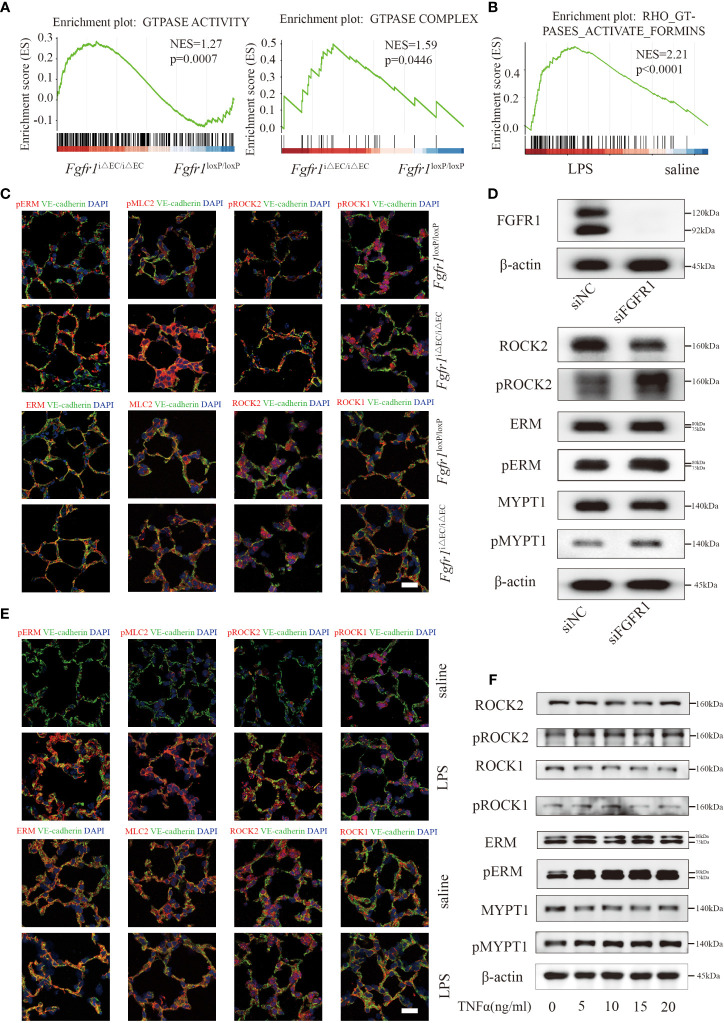
Endothelial ROCK2 was activated in ALI/ARDS model and functioned as a downstream of FGFR1. RNA-seq analysis was performed on ECs isolated from LPS-treated *Fgfr1* ^iΔEC/iΔEC^ mice and *Fgfr1* ^loxP/loxP^ mice. Gene set enrichment analysis of differentially expressed genes showed enrichment of GTPase activity and the GTPase complex **(A)**, which are related to RhoA activation. RhoA GTPase activation was enriched by GSEA in the LPS group compared with the saline group **(B)**. Endothelial ROCK2 activity was assessed by the colocalization of pERM, ERM, pMLC2, MLC2, pROCK2, ROCK2, pROCK1 and ROCK1 (red fluorescent protein) with VE-cadherin (green fluorescent protein) by immunofluorescent staining. Representative images of immunofluorescent staining of endothelial ROCK2 activity in the *Fgfr1* ^iΔEC/iΔEC^ mice vs the *Fgfr1* ^loxP/loxP^ mice group **(C)** and the saline vs LPS group **(E)** are shown. n=3 per group. Scale bars: 40 µm. HUVECs were transfected with siNC or siFGFR1 for 48 h. The expression of FGFR1 after siRNA transfection was detected by WB with FGFR1 and β-actin antibodies. The phosphorylation of ERM, MYPT1 and ROCK2 was assessed **(D)**. In addition, HUVECs were treated with 0, 5, 10, 15 and 20 ng/ml of TNFα for 12 h, and ROCK2 activity was assessed by WB **(F)**.

Next, we sought to unravel the downstream molecular mechanisms of endothelial FGFR1 involved in vascular leakage and inflammation. As a result, we subjected pulmonary ECs from *Fgfr1* ^iΔEC/iΔEC^ mice and *Fgfr1* ^loxP/loxP^ mice to bulk RNA sequencing. By GSEA, the *Fgfr1* ^iΔEC/iΔEC^ mouse group was enriched for increased GTPase activity-related terms (**Figure 3A**), consistent with the increased Rho GTPase activity found in our RNA-seq of the saline and LPS groups (**Figure 3B**) and previous studies (5, 25). We examined endothelial ROCK activation which correlated with the ratio of pERM/ERM and pMLC2/MLC2 in ECs, suggesting robust ROCK activation in *Fgfr1* ^iΔEC/iΔEC^ mice (**Figure 3C**). It is well known that RhoA GTPase has two isoforms downstream effectors, ROCK1 and ROCK2 (26). According to previous studies, ROCK1 and ROCK2 exert redundant functions in the regulation of actomyosin contractility in mouse embryonic fibroblasts (27). In order to elucidate the effect of each isoform of ROCKs, the autophosphorylation expression levels of pROCK1 (S1333) and pROCK2 (S1366) in ECs were assessed by immunofluorescent staining. We found that pROCK2 was significantly increased in *Fgfr1* ^iΔEC/iΔEC^ mice and that pROCK1 showed no obvious change (**Figure 3C**). Furthermore, knockdown of FGFR1 with siRNA FGFR1 without TNFα treatment significantly increased ROCK2 activity in HUVECs (**Figures 3D**; **S3A**) and the effect of siRNA FGFR1 transfection was tested by western blotting (**Figures 3D**, **S3A**). Similarly, endothelial ROCK2 was activated in WT mice and HUVECs challenged with different doses of TNFα (**Figures 3E, F**, **S3B**). Collectively, these data suggested that endothelial ROCK2 functioned as a downstream of FGFR1 and was activated in the lungs of ALI/ARDS mice and TNFα-stimulated HUVECs.

### AAV Vec-tie shROCK2 effectively attenuated inflammation and vascular leakage in *Fgfr1* ^iΔEC/iΔEC^ mice

**Figure 4:**
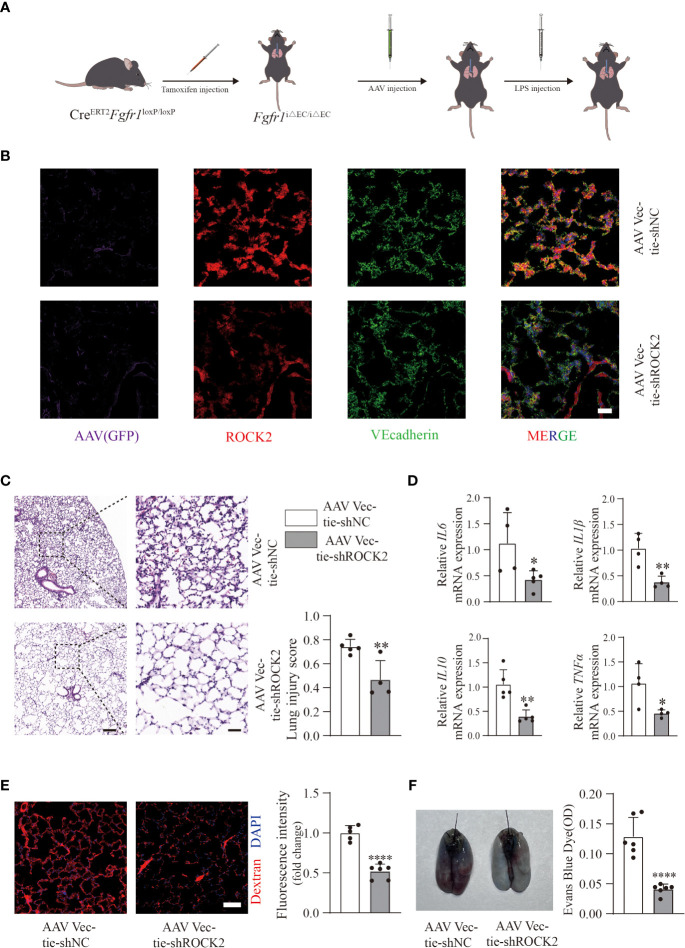
AAV Vec-tie-shROCK2 effectively inhibited inflammation and vascular leakage in *Fgfr1* ^iΔEC/iΔEC^ mice. After intraperitoneal injection of tamoxifen, *Fgfr1* ^iΔEC/iΔEC^ mice were intratracheally injected with AAV Vec-tie-shROCK2 or AAV Vec-tie-shNC. One month later, they were intratracheally injected with LPS (2 mg/kg) **(A)**. The efficiency of AAV Vec-tie expressing ROCK2-specific shRNA in pulmonary ECs was verified by immunofluorescence. Representative images of immunofluorescent staining of GFP, ROCK2 and VE-cadherin in lung tissues from *Fgfr1* ^iΔEC/iΔEC^ mice treated with GFP-AAV Vec expressing either negative control shRNA or ROCK2 specific shRNA are shown **(B)**. n=3 per group. Scale bars: 50 µm. The AAV Vec-tie-shROCK2 group showed significantly decreased inflammation. Representative images stained with hematoxylin and eosin and lung injury scores are shown **(C)**. n=4 or 5 per group. Scale bars: 200 µm (left) and 40 µm (right). Relative mRNA expression levels of inflammatory cytokines (*IL6*, *IL1β*, *IL10*, *TNFα*) were examined by RT-PCR **(D)**. n=4 or 5 per group. Vascular permeability was also significantly decreased in the AAV Vec-tie-shROCK2 group. Representative images of immunofluorescent staining and quantitative mean fluorescence intensity of AF555-dextran are shown **(E)**. n=5 or 6 per group. Scale bars: 50 µm. Representative images of the gross appearance of EBD-stained lungs and quantification of EBD content in the lungs are shown **(F)**. n=6 per group. Each bar represents the mean ± SD; *p < 0.05, **p < 0.01 and ****p < 0.0001.

To precisely clarify the contribution of endothelial ROCK2 in LPS-treated *Fgfr1* ^iΔEC/iΔEC^ mice, we intratracheally injected AAV Vec-tie-shROCK2 or AAV Vec-tie-shNC into *Fgfr1* ^iΔEC/iΔEC^ mice after tamoxifen induction. One month later, LPS (2 mg/kg) was intratracheally injected (**Figure 4A**). The knockdown efficiency of AAV Vec-tie-shROCK2 was verified by immunofluorescence (**Figure 4B**). Histology (**Figure 4C**) and inflammatory cytokines (**Figure 4D**) showed a significant decline in inflammation in *Fgfr1* ^iΔEC/iΔEC^ mice treated with AAV Vec-tie-shROCK2. Moreover, decreased extravasation of AF555-dextran (**Figure 4E**) and EBD (**Figure 4F**) demonstrated that knockdown of endothelial ROCK2 effectively blocked vascular leakage in *Fgfr1* ^iΔEC/iΔEC^ mice. Overall, our data indicated that inhibition of endothelial ROCK2 activity exhibited an effective way to relieve the aggravation due to lack of endothelial FGFR1 and provided further evidence that deficiency of endothelial FGFR1 contributed to inflammation and vascular permeability *via* activation of ROCK2. Furthermore, this finding provided an effective and therapeutic target for ALI/ARDS.

### TDI01 effectively attenuated ALI/ARDS in *Fgfr1* ^iΔEC/iΔEC^ mice and endothelial dysfunction in FGFR1-deficient HUVECs

**Figure 5:**
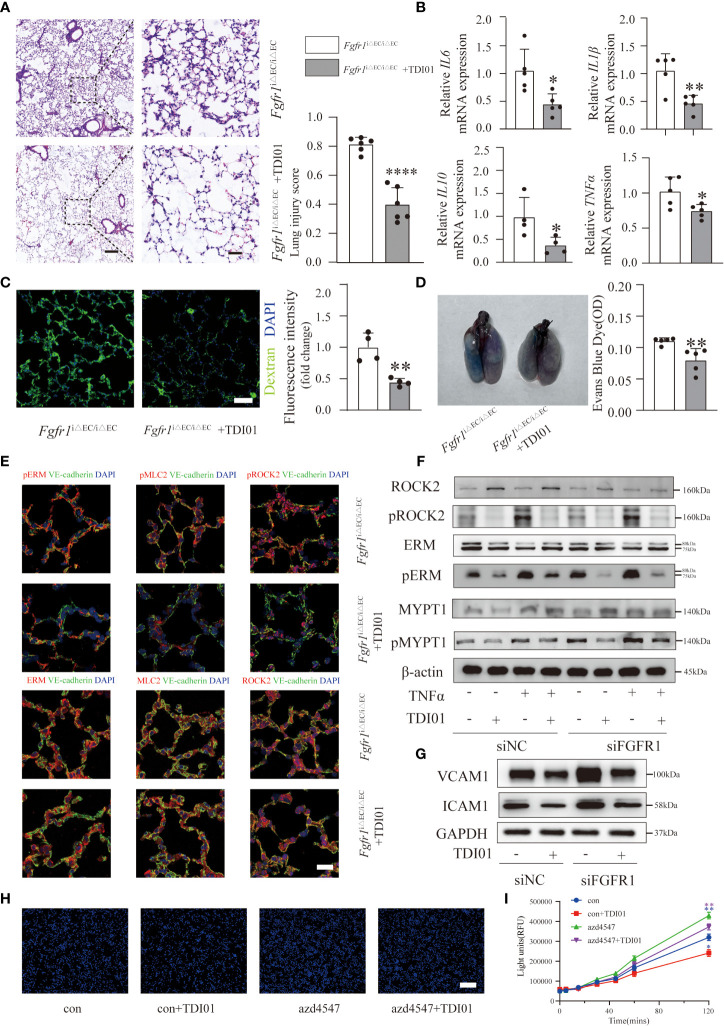
TDI01 effectively attenuated ALI/ARDS in *Fgfr1* ^iΔEC/iΔEC^ mice and endothelial dysfunction in FGFR1-deficient HUVECs. Inhibition of ROCK2 by TDI01 suppressed the inflammatory response of *Fgfr1* ^iΔEC/iΔEC^ mice. Representative images stained with hematoxylin and eosin and lung injury scores are shown **(A)**. n=6 per group. Scale bars: 200 µm (left) and 40 µm (right). The relative mRNA expression of inflammatory cytokines (*IL6*, *IL1β*, *IL10*, *TNFα*) was reduced in the TDI01-pretreated *Fgfr1* ^iΔEC/iΔEC^ group **(B)**, n=4 or 5 per group. Vascular barrier dysfunction in *Fgfr1* ^iΔEC/iΔEC^ mice was rescued by TDI01, as indicated by decreased leakage of FITC-dextran **(C)**, n=4 per group, scale bars: 50 µm and EBD **(D)**, n=5 per group. Endothelial ROCK2 activity was inhibited by TDI01 in *Fgfr1* ^iΔEC/iΔEC^ mice and was assessed by the colocalization of pERM, ERM, pMLC2, MLC2, pROCK2 and ROCK2 (red fluorescent protein) with VE-cadherin (green fluorescent protein) by immunofluorescent staining. Representative images are shown **(E)**. n=5 per group. Scale bars: 40 µm. HUVECs were transfected with siNC or siFGFR1 and pretreated with TDI01 (0, 10 µM) for 24 h, and then treated with or without 20 ng/ml of TNFα for 12 h. ROCK2 activity was determined by the phosphorylation of ERM, MYPT1 and ROCK2 **(F)**. HUVECs were transfected with siNC or siFGFR1 and pretreated with TDI01 (0, 10 µM) for 24 h, and then treated with 20 ng/ml of TNFα for 12 h. The expression of VCAM1 and ICAM1 was assessed by WB **(G)**. HUVECs were pretreated with TDI01 (0, 1 µM) for 24 h, treated with or without azd4547 (1 µM) for 12 h and then challenged by 20 ng/ml of TNFα for 12 h. Hoechst 33342-labeled monocytes were added to each well and co-incubated for 4 h. Representative images of adhered Hoechst 33342-labeled monocytes are shown **(H)**. n=3 per group, scale bars: 200 µm. HUVECs were pretreated with TDI01 (0, 1 µM) for 24 h, and treated with or without azd4547 (1 µM) for 12 h and then were plated for permeability assay. FITC-dextran and TNFα (20 ng/ml) were simultaneously administered into HUVECs and the fluorescence intensity were recorded at 0, 5, 15, 30, 45, 60, 120 min **(I)**. n=3 per group. Each bar represents the mean ± SD; *p < 0.05, **p < 0.01 and ****p < 0.0001.

After demonstrating the effects of activated ROCK2, we selected ROCK2 rather than ROCK1 as the therapeutic target for ALI/ARDS in *Fgfr1* ^iΔEC/iΔEC^ mice. TDI01 is a highly selective ROCK2 inhibitor and has shown good efficacy (Beijing Tide Pharmaceutical Co., Ltd.). Therefore, we pretreated *Fgfr1* ^iΔEC/iΔEC^ mice with TDI01 (200 mg/kg/day) for 3 days before injection of LPS. Subsequently we compared the histology, inflammatory response and pulmonary vascular leakage. The results showed that TDI01 significantly alleviated pulmonary edema and inflammatory response based on significantly decreased lung injury scores (**Figure 5A**) and inflammatory cytokines (**Figure 5B**). Moreover, the extravasation of FITC-dextran (**Figure 5C**) and EBD (**Figure 5D**) was reduced, suggesting that TDI01 protected against pulmonary vascular leakage. Mechanistically, the TDI01-pretreated *Fgfr1* ^iΔEC/iΔEC^ mouse group showed decreased pERM, pMLC2 and pROCK2 (**Figure 5E**). In addition, western blotting analysis indicated that knockdown of FGFR1 by siRNA FGFR1 in HUVECs with or without TNFα stimulation enhanced the ratio of pROCK2/ROCK2, pERM/ERM and pMYPT1/MYPT1 (**Figures 5F**, **S4A**). Importantly, TDI01 significantly downregulated the phosphorylation of ROCK2,ERM and MYPT1 (**Figures 5F**, **S4A**), demonstrating that TDI01 inhibited ROCK2 activation *in vitro*. When insulted by inflammatory mediators such as TNFα and LPS, ECs transform into a dysfunctional phenotype with increased adhesion molecules (such as ICAM1, VCAM1) on the cell surface to adhere to leukocytes and increased permeability (2, 28). To investigate whether knockdown of FGFR1 and ROCK2 activation in HUVECs affect the expression of ICAM1 and VCAM1, HUVECs were transfected with siRNA FGFR1 with or without pretreatment with TDI01 for 24 h before stimulation with TNFα for 12 h. The results suggested that knockdown of FGFR1 enhanced the expression of ICAM1 and VCAM1, which was dampened by inhibition of its downstream ROCK2 activity (**Figures 5G**, **S4B**). We also performed a monocyte adhesion assay to assess the recruitment of inflammatory cells to HUVECs. HUVECs were pretreated with TDI01 for 24 h with or without azd4547 (FGFR pan inhibitor) for 12 h, and then challenged by TNFα for 12 h. THP-1 cells were labeled with Hoechst 33342 and then co-incubated with HUVECs. A significant increase in the recruitment of monocytes was observed in the azd4547-treated group, which was blocked by TDI01 (**Figures 5H**, **S4C**). To determine the role of decreased FGFR1 and ROCK2 activation in regulating vascular permeability, *in vitro* TNFα-stimulated HUVECs were pretreated with TDI01 for 24 h and then treated with or without azd4547 for 12 h. The fluorescence intensity of extravasated FITC-dextran was measured (**Figure 5I**) and the results indicated that loss of FGFR1 signaling promoted vascular leakage, which was blocked by inhibition of ROCK2 activity. Taken together, loss of FGFR1 and activated ROCK2 made HUVECs more adhesive to inflammatory cells and aggravated vascular leakage. Meanwhile, TDI01 inhibited ROCK2 activity and played a therapeutic role.

### TDI01 inhibited ROCK2 activity in LPS-induced ALI/ARDS and TNFα-stimulated HUVECs

**Figure 6:**
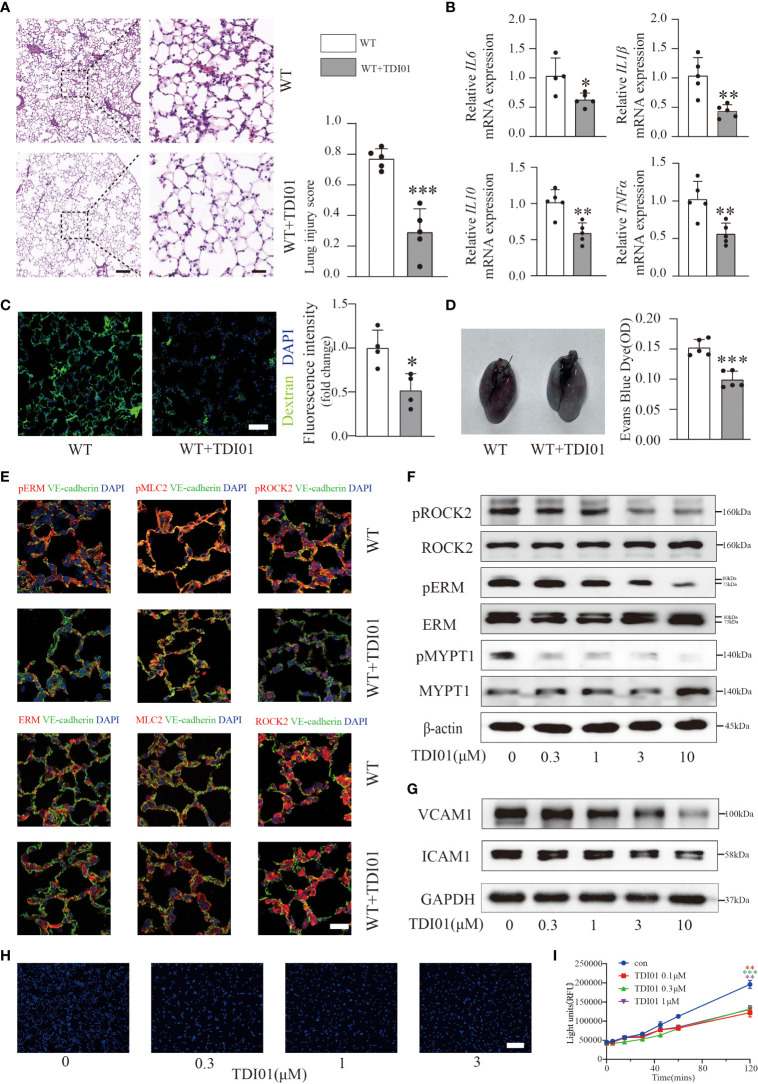
TDI01 could rescue LPS-induced ALI/ARDS and TNFα-stimulated HUVECs. Representative lung sections were stained with hematoxylin and eosin, and lung injury scores were quantified, n=5 per group. Scale bars: 200 µm (left) and 40 µm (right) **(A)**. The relative mRNA expression of inflammatory cytokines (*IL6*, *IL1β*, *IL10*, *TNFα*) in the TDI01-pretreated WT group was reduced **(B)**. n=4 or 5 per group. Vascular permeability was tested by FITC-dextran **(C)** and EBD **(D)**, and TDI01 has a protective role in the vascular barrier, as evidenced by decreased extravasation of FITC-dextran **(C)**, n=4 per group, scale bars: 50 µm and Evans blue dye **(D)**, n=5 per group. Endothelial ROCK2 activity was inhibited by TDI01 in WT mice and was assessed by the colocalization of pERM/ERM, pMLC2/MLC2 and pROCK2/ROCK2 (red fluorescent protein) with VE-cadherin (green fluorescent protein) by immunofluorescent staining. Representative images are shown **(E)**. n=3 per group, scale bars: 20 µm. HUVECs were pretreated with 0, 0.3, 1, 3 and 10 µM of TDI01 for 24 h and then treated with 20 ng/ml of TNFα for 12 h. ROCK2 activity was examined by WB **(F)**. The expression of VCAM1 and ICAM1 was assessed by WB **(G)**. HUVECs were pretreated with TDI01 (0, 0.3, 1, 3 µM) for 24 h and then challenged by 20 ng/ml of TNFα for 12 h. Hoechst 33342-labeled monocytes were added to each well and co-incubated for 4 h. Representative images of adhered Hoechst 33342-labeled monocytes are shown **(H)**. n=3 per group, scale bars: 200 µm. HUVECs were pretreated with TDI01 (0, 0.1,0.3,1 µM) for 24 h, stimulated with TNFα (20 ng/ml) for 12 h and then plated for permeability assay. The fluorescence intensity of FITC-dextran was recorded at 0, 5, 15, 30, 45, 60, 120 min **(I)**. n=3 per group. Each bar represents the mean ± SD; *p < 0.05, **p < 0.01 and ***p < 0.001.

Previous studies have noted that RhoA GTPase and its effectors (ROCK1, ROCK2) inhibitors offer promising targets for ALI/ARDS (20). To test whether TDI01 still has a protective role in more severe conditions, we performed pretreatment with TDI01 (200 mg/kg/day) for 3 days before injection of LPS and used a higher dose of LPS (10 mg/kg) to obtain an ALI/ARDS model with more severe inflammation and vascular permeability. Histology (**Figure 6A**) and inflammatory cytokine expression (**Figure 6B**) demonstrated an evident reduction in inflammation in the TDI01-pretreated group. In terms of its effect on vascular permeability, we also observed a significant decrease in exosmic FITC-dextran (**Figure 6C**) and EBD (**Figure 6D**), which suggested that TDI01 could exert its function in the preservation of vascular integrity. Endothelial ROCK2 activity was significantly inhibited *in vivo* (**Figure 6E**). Furthermore, HUVECs were pretreated with TDI01 before TNFα challenge and western blotting analysis showed that ROCK2 activity (**Figures 6F**, **S5A**), ICAM1 and VCAM1 (**Figures 6G**, **S5B**) were inhibited in a dose-dependent manner, and TDI01 effectively attenuated TNFα-induced monocyte adhesion (**Figures 6H**, **S5C**). TDI01 also protected HUVECs against TNFα-induced vascular leakage (**Figure 6I**). Overall, TDI01 exhibited promising effectiveness for the treatment of ALI/ARDS.

## Discussion

In the present study, we demonstrated that LPS triggered a significant increase in inflammation as well as pulmonary vascular leakage and that endothelial cells acting as a semipermeable barrier played a critical role in the progression of ALI/ARDS. We showed that endothelial FGFR1 signaling was significantly reduced in the LPS-treated WT mouse group. We therefore utilized *Fgfr1* ^iΔEC/iΔEC^ mice to conditionally knock out endothelial FGFR1 and confirmed that endothelial ROCK2, but not endothelial ROCK1, was activated in *Fgfr1* ^iΔEC/iΔEC^ mice. Meanwhile, *in vitro* knockdown of FGFR1 by siRNA activated ROCK2, leading to endothelial dysfunction with increased adhesive properties and permeability in TNFα-treated HUVECs. Furthermore, inhibition of endothelial ROCK2 by AAV Vec-tie-shROCK2 significantly attenuated inflammation and alleviated vascular leakage in *Fgfr1* ^iΔEC/iΔEC^ mice. In addition, we found that TDI01, a novel selective ROCK2 inhibitor, strongly inhibited ROCK2 activity in LPS-induced ALI/ARDS *in vivo* and TNFα-treated HUVECs *in vitro*, suggesting promising therapeutic prospects for ALI/ARDS.

Healthy endothelial cells exhibit an anti-inflammatory phenotype to regulate inflammation. However, when stimulated by LPS, damage-associated molecular patterns (DAMPs) and cytokines, they will transform into an inflammatory phenotype, prone to become vascular leaky and attractive to inflammation (2, 4). Thus, in this study, we focused on pulmonary endothelial cells and sorted pulmonary ECs from saline or LPS-treated WT mice through magnetic beads to determine transcriptional changes. Our data demonstrated that endothelial FGFR1 was significantly decreased in the LPS-treated WT mouse group. Endothelial FGFR1 has been reported to be an essential element for maintaining vascular homeostasis (11). However, its mechanism in ALI/ARDS has not yet been elucidated. We next sought to explore the role of endothelial FGFR1 in regulating inflammation and pulmonary vascular permeability. Therefore, we constructed *Fgfr1* ^iΔEC/iΔEC^ mice with conditional knockout of endothelial FGFR1 for further experiments. A recent study showed that administration of recombinant FGF2 alleviated pulmonary vascular leakage and attenuated the inflammatory response in sepsis-induced ALI by stabilizing adherens junctions (29). Additionally, FGF10 exhibited its protective function in preservation of alveolar-capillary barrier integrity in high altitude pulmonary edema (30). The similarity among these studies suggests a protective role of FGFs and is consistent with our finding of aggravated inflammation and pulmonary vascular permeability in *Fgfr1* ^iΔEC/iΔEC^ mice compared with *Fgfr1* ^loxP/loxP^ mice. We then sought to identify the downstream targets of FGFR1 and performed RNA-seq analysis of pulmonary ECs from LPS-treated *Fgfr1* ^iΔEC/iΔEC^ mice and *Fgfr1* ^loxP/loxP^ mice. Strikingly, our data confirmed that endothelial ROCK2 rather than endothelial ROCK1 was activated not only in *Fgfr1* ^iΔEC/iΔEC^ mice but also in LPS-treated WT mice, as evidenced by the increased phosphorylation of a variety of substrates, such as the ERM, MYPT1, MLC and autophosphorylation by ROCK2 on S1366, while *in vitro* knockdown of FGFR1 in HUVECs also showed increased ROCK2 activity. In turn, this finding is consistent with the report that exogenous FGF treatment dependent on FGFR-induced activation of PI3K-Akt-Rac1 signaling inhibits RhoA activity and protects the blood-brain barrier after intracerebral hemorrhage in mice (31). We also found that decreased FGFR1 and ROCK2 activation made TNFα-treated HUVECs more adhesive to inflammatory cells and more permeable. Overall, our data provided further evidence that deletion of endothelial FGFR1 signaling by upregulating the ROCK2 activity-mediated pathway led to exacerbation of ALI/ARDS.

To determine the role of ROCK2 in LPS-treated *Fgfr1* ^iΔEC/iΔEC^ mice, we intratracheally injected AAV Vec-tie-shROCK2 for the precise knockdown of ROCK2 in pulmonary ECs. Our data showed that knockdown of ROCK2 significantly alleviated inflammation and vascular leakage in LPS-treated *Fgfr1* ^iΔEC/iΔEC^ mice and identified endothelial ROCK2 as a promising therapeutic target. Notably, RhoA and its downstream effectors ROCKs (ROCK1 and ROCK2) inhibit dephosphorylation of MLCP and directly phosphorylate MLC to augment the phosphorylation of MLC, resulting in endothelial contraction and subsequent vascular barrier dysfunction (5). Constructional vascular integrity is supported by tight junctions and adherens junctions, which are linked to the endothelial actin cytoskeleton (4). Disruption of the endothelial barrier is attributed to the activation of the actin-myosin contractile apparatus, which is regulated by the level of myosin light chain (MLC) phosphorylation. Indeed, phosphorylation is dynamic and regulated by the interaction between calcium/calmodulin-dependent MLC kinase (MLCK, phosphorylation) and MLC phosphatase (MLCP, dephosphorylation) (5, 32). When MLC is excessively phosphorylated, the tension of actinomyosin contraction pulls endothelial cells apart and contributes to the formation of intercellular gaps.

Due to the extensive expression and complexity of FGF/FGFR1 signaling, we sought a more specific drug for treatment to prevent unwanted side effects. TDI01 is a novel and highly selective ROCK2 inhibitor that is currently being tested in phase I clinical trials for the treatment of idiopathic pulmonary fibrosis and silicosis. Compared with nonselective inhibitors such as Y27632 and fasudil, which target both ROCK1 and ROCK2 (20), TDI01 is expected to have a greater therapeutic effect and fewer side effects. Our data demonstrated that TDI01 protected against inflammation and vascular leakage in LPS-induced ALI/ARDS. In addition, TDI01 rescued endothelial dysfunction by downregulating the expression of VCAM1 and ICAM1, attenuating inflammatory cell adhesion and decreasing permeability. The present study used pretreatment before LPS injection, and TDI01 successfully achieved good preventative and curative effects in alleviating vascular leakage and inflammation, indicating its potential for clinical translation.

In summary, our findings suggest that endothelial FGFR1 signaling plays a key role in preserving the integrity of the pulmonary vascular barrier, and reveal that activated ROCK2 contributing to EC contraction and gap formation of neighboring ECs is responsible for the exacerbation of ALI/ARDS in *Fgfr1* ^iΔEC/iΔEC^ mice. We also found that TDI01, a new selective ROCK2 inhibitor, prevents and treats ALI/ARDS and provides potential valuable insights for the treatment of this disease.

## Data availability statement

The datasets presented in this study can be found in online repositories. The names of the repository/repositories and accession number(s) can be found below: GSE216635 (GEO).

## Ethics statement

The animal study was reviewed and approved by the committees of West China Second University Hospital, Sichuan University.

## Author contributions

This study was designed and drafted by YH, HW, B-SD and CW. YD performed all the experiments and prepared the manuscript. XH performed RNA-seq analysis. WZ analyzed the data. HZ and CM revised the manuscript. All authors contributed to the article, read, and approved the final manuscript. All authors contributed to the article and approved the submitted version.
