# Supplementary material for: Deficiency of endothelial FGFR1 signaling via upregulation of ROCK2 activity aggravated ALI/ARDS
Source: Front Immunol. 2023 Mar 10;14:1041533. doi: 10.3389/fimmu.2023.1041533 (PMC10036754; doi:10.3389/fimmu.2023.1041533)
Supplement: Supplementary file 1 [file DataSheet_1.docx]

**Supplementary Materials**


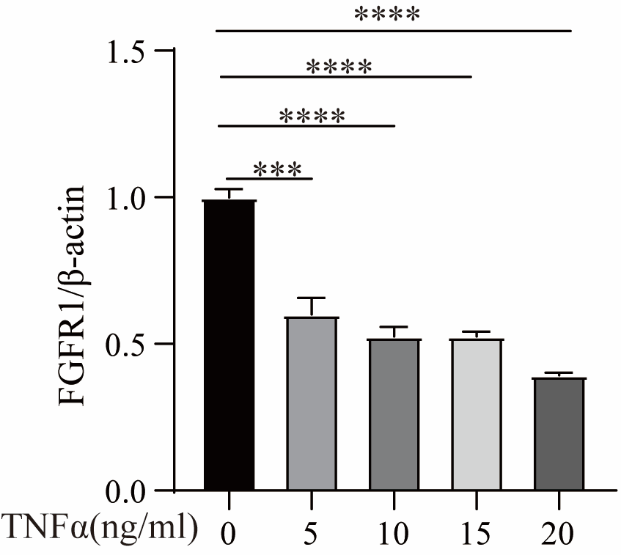


**Figure S1.** (Related to Fig. 1). HUVECs were treated with 0, 5, 10, 15 and 20 ng/ml TNFα for 12 h. The relative densities of FGFR1 in HUVECs which were stimulated by TNFα for 12 h were quantified. n=3 per group. Each bar represents the mean ± SD; *** p < 0.001 and **** p < 0.0001.


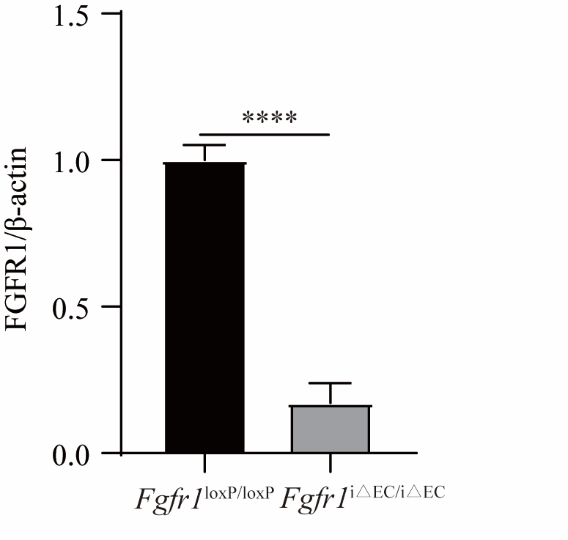
**Figure S2.** (Related to Fig. 2). Pulmonary ECs were isolated from *Fgfr1*^loxP/loxP^ mice and *Fgfr1*^iΔEC/iΔEC^ mice. The relative densities of FGFR1 were quantified. n=3 per group. Each bar represents the mean ± SD; **** p < 0.0001.


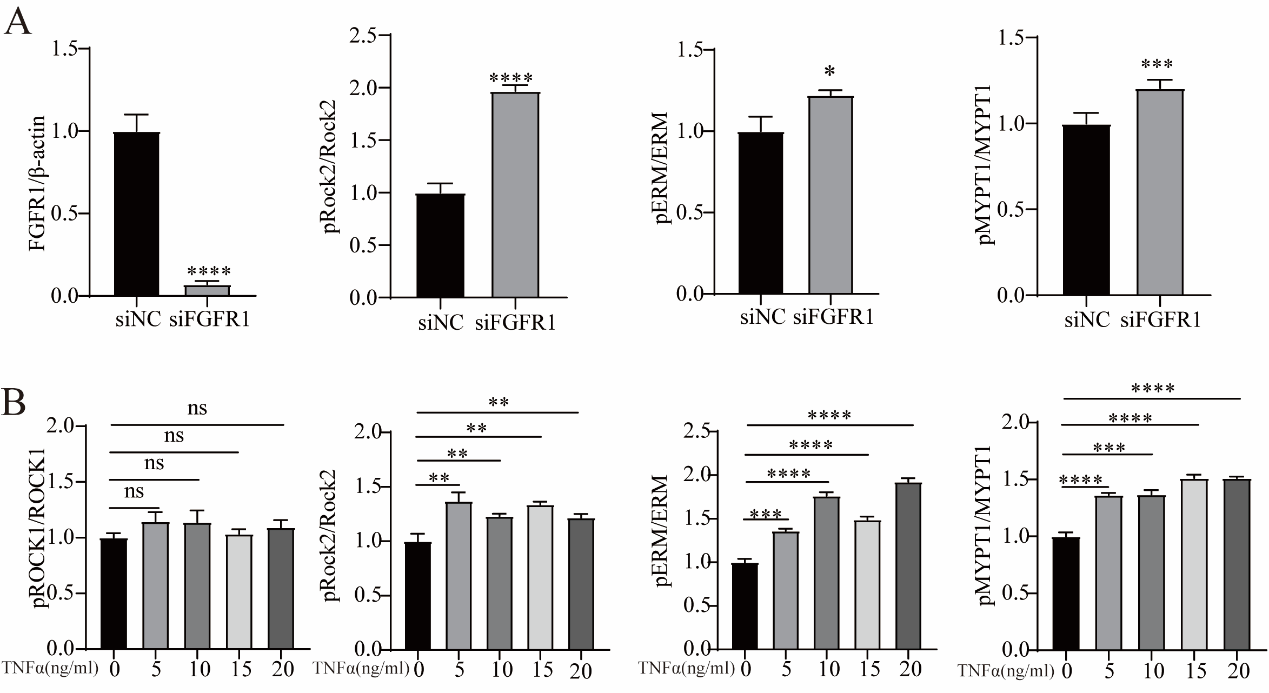


**Figure S3.** (Related to Fig. 3). The relative densities of FGFR1 in HUVECs which were transfected by siNC and siFGFR1 were quantified (A). Densitometric quantification of pROCK2/ROCK2, pERM/ERM and pMYPT1/MYPT1(A) at 48 h after siRNA transfection was shown. n=3 per group. HUVECs were treated with 0, 5, 10, 15 and 20 ng/ml TNFα for 12 h. Relative densities of western blotting images were quantified and the ratio of pROCK1/ROCK1, pROCK2/ROCK2, pERM/ERM and pMYPT1/MYPT1 was shown (B). n=3 per group. Each bar represents the mean ± SD; ns>0.05, *p < 0.05, **p < 0.01, *** p < 0.001 and **** p < 0.0001.

**
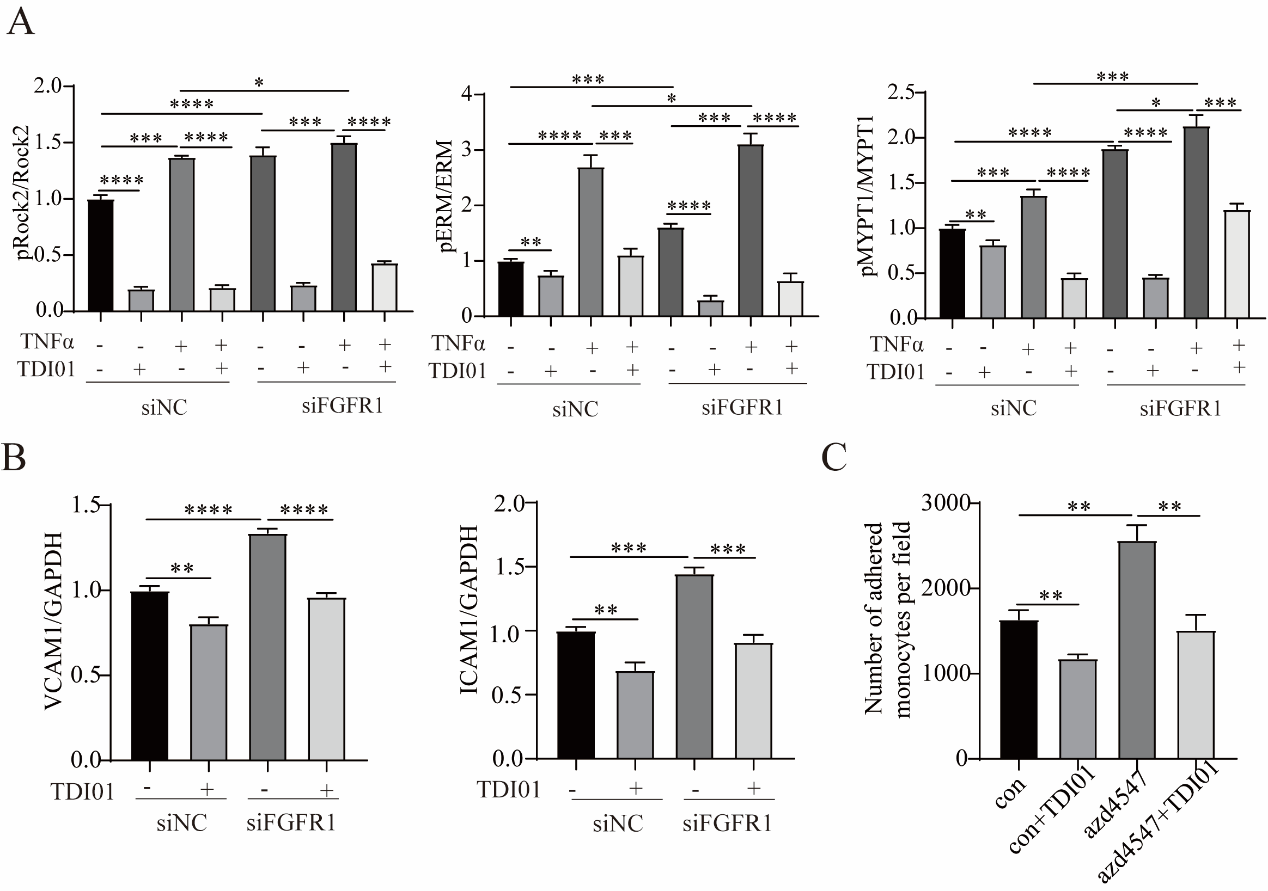
Figure S4.** (Related to Fig. 5). HUVECs were transfected with siNC or siFGFR1 and pretreated with TDI01 (0, 10 µM) for 24 h, and then treated with or without 20 ng/ml TNFα for 12 h. Densitometric quantification of pROCK2/ROCK2, pERM/ERM and pMYPT1/MYPT1(A) was shown. n=3 per group. HUVECs were transfected with siNC or siFGFR1 and pretreated with TDI01 (0, 10 µM) for 24 h, and then treated with 20 ng/ml TNFα for 12 h. The relative densities of VACM1 and ICAM1 in HUVECs which were quantified (B). n=3 per group. HUVECs were pretreated with TDI01(0, 1 µM) for 24 h with or without azd4547(1 µM) for 12 h, and then challenged by 20 ng/ml TNFα for 12 h. Hoechst 33342 labeled monocytes were added to each well, co-incubated for 4 h and then were counted to evaluate the endothelial adherence capacity (C). Each bar represents the mean ± SD; *p < 0.05, **p < 0.01, *** p < 0.001 and **** p < 0.0001.


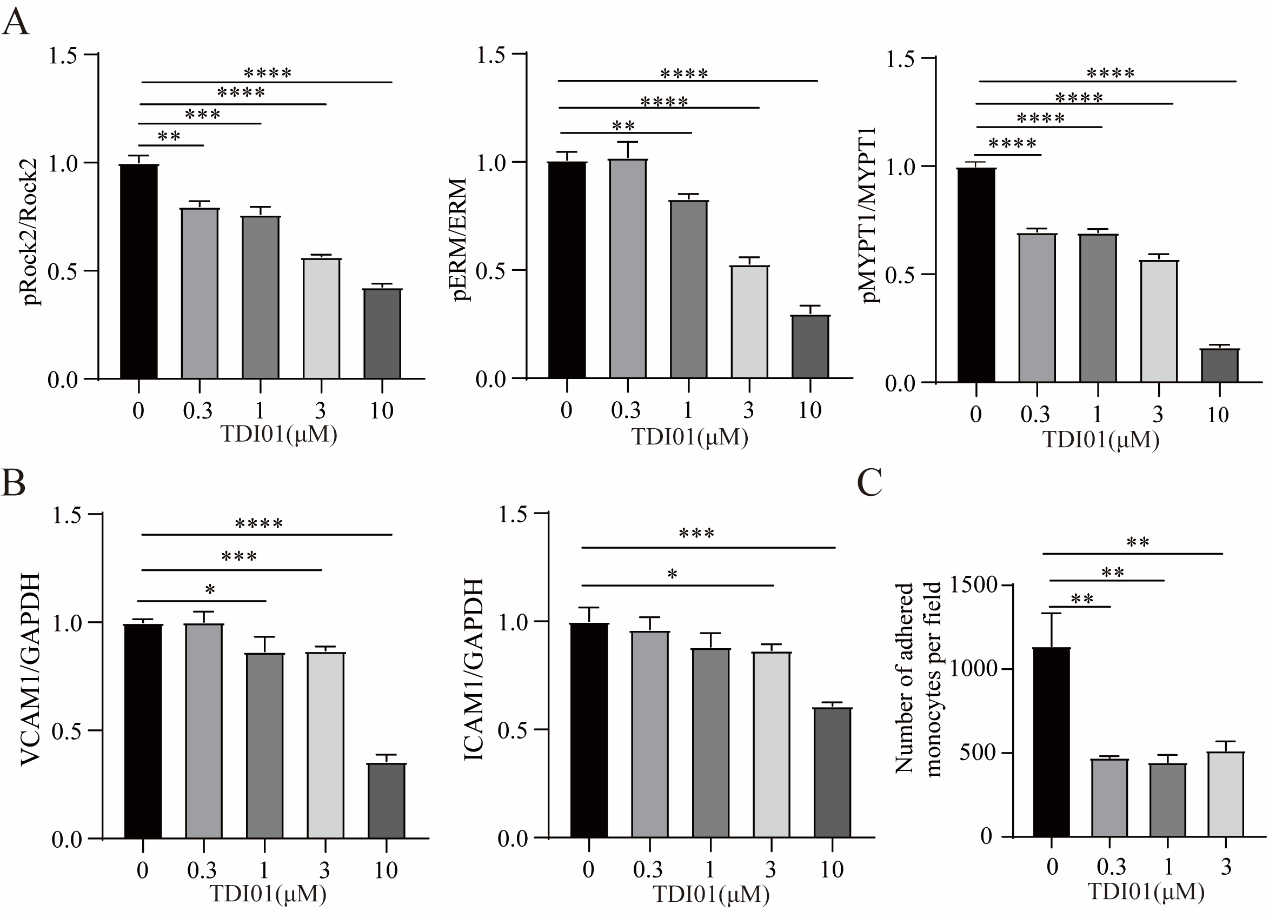


**Figure S5.** (Related to Fig. 6). HUVECs were pretreated with 0, 0.3, 1, 3 and 10 µM TDI01 for 24 h and then treated with 20 ng/ml TNFα for 12 h. Densitometric quantification of pROCK2/ROCK2, pERM/ERM, pMYPT1/MYPT1 (A), VCAM1/GAPDH and ICAM1/GAPDH (B) was shown. n=3 per group. HUVECs were pretreated with 0, 0.3, 1, 3 µM TDI01 for 24 h and then challenged by 20 ng/ml TNFα for 12 h. Hoechst 33342 labeled monocytes were added to each well, coincubated for 4 h and then were counted to evaluate the endothelial adherence capacity (C) Each bar represents the mean ± SD; *p < 0.05, **p < 0.01, *** p < 0.001 and **** p < 0.0001.

**Table S1 Sequences of AAV Vec-tie-shROCK2 and AAV Vec-tie-shNC**

| AAV Vec-tie-shROCK2 | |
| --- | --- |
| Forward | AAGGTATATTGCTGTTGACAGTGAGCGGCATCTCTTGAAGAAACAAATTAGTGAAGCCACAGATGTAATTTGTTTCTTCAAGAGATGCTGCCTACTGCCTCG |
| Reverse | CGAGGCAGTAGGCAGCATCTCTTGAAGAAACAAATTACATCTGTGGCTTCACTAATTTGTTTCTTCAAGAGATGCCGCTCACTGTCAACAGCAATATACCTT |
| AAV Vec-tie-shNC | |
| Forward | GATCCGTTCTCCGAACGTGTCACGTAATTCAAGAGATTACGTGACACGTTCGGAGAATTTTTTC |
| Reverse | AATTGAAAAAATTCTCCGAACGTGTCACGTAATCTCTTGAA  TTACGTGACACGTTCGGAGAACG |

**Table S2 Primers used in quantitative real-time PCR**

| **Gene** | **Primer sequence** | |
| --- | --- | --- |
| GAPDH | Forward | 5’- AGGTCGGTGTGAACGGATTTG -3’ |
|  | Reverse | 5’- TGTAGACCATGTAGTTGAGGTCA -3’ |
| IL6 | Forward | 5’-GCCTTCTTGGGACTGATGCT-3’ |
|  | Reverse | 5’-TGTGACTCCAGCTTATCTCTTGG-3’ |
| IL1β | Forward | 5’-TGCCACCTTTTGACAGTGATG-3’ |
|  | Reverse | 5’-AAGGTCCACGGGAAAGACAC-3’ |
| IL10 | Forward | 5’-ACTACCAAAGCCACAAGGCA-3’ |
|  | Reverse | 5’-ACACCTTGGTCTTGGAGCTTATTA-3’ |
| TNFα | Forward | 5’-CCCTCACACTCAGATCATCTTCT-3’ |
|  | Reverse | 5’-GCTACGACGTGGGCTACAG-3’ |
| FGFR1 | Forward | 5’- AGCTCCCTACTGGACATCCC -3’ |
|  | Reverse | 5’- AGGTGGCATAGCGAACCTTG -3’ |

**Table S3 siRNA primer sequences**

| siFGFR1 | |
| --- | --- |
| Forward | GGAUGAUGAUGAUGAUGAU |
| Reverse | AUCAUCAUCAUCAUCAUCC |
| siNC | |
| Forward | UUCUCCGAACGUGUCACGUTT |
| Reverse | ACGUGACACGUUCGGAGAATT |
